# Reflex seizures induced by micturition: a case report

**DOI:** 10.1186/s42494-022-00107-y

**Published:** 2023-01-13

**Authors:** Zhiyun Zhang, Qiwei Li, Tiejia Jiang, Jiajia Fang

**Affiliations:** 1 Department of Neurology, The Fourth Affiliated Hospital, International Institutes of Medicine, Zhejiang University School of Medicine, Yiwu, 322000 China; 2 Department of Neurology, The Children’s Hospital of Zhejiang University School of Medicine, Hangzhou, 310009 China

**Keywords:** Reflex seizure, Micturition, Scalp electroencephalogram, Ictogenesis, Case report

## Abstract

**Background:**

Reflex seizures (RS) induced by micturition are extremely rare, and the clinical and electroencephalogram features of RS are not widely known among clinicians. In particular, the origin of the epileptic area is still unclear.

**Case presentation:**

An 8-year-old girl who had generalized tonic-clonic seizures was diagnosed with RS induced by micturition based on the clinical manifestation and EEG recordings. We also reviewed the clinical and EEG characteristics of RS induced by micturition in literature by searching the databases of PubMed and MEDLINE using keywords “micturition reflex seizure”, “reflex seizure induced by micturition”, and “micturition induced seizure” by January 2022. We speculate that the mechanism of micturition-induced RS may involve excessive neuronal excitation in regions that participate in micturition.

**Conclusions:**

The RS in this patient was considered to be induced by micturition. Awareness should be raised to this rare form of RS among practitioners.

## Background

Reflex seizures (RS) are provoked seizures by specific sensory stimuli, such as visual precipitants, eating, bathing, and writing. In 1964, Zivin et al. first reported a 14-year-old male patient with RS induced by micturition [1], and few studies have reported it subsequently. Due to the quite small number of known cases, little is known about the clinical presentation and electroencephalogram (EEG) features of RS induced by micturition. In this report, we describe a pediatric case of micturition-induced focal epilepsy diagnosed by ictal EEG recording. We also review other cases of micturition-induced RS in literature and summarize its main clinical and EEG characteristics, in order to improve awareness among practitioners about this rare form of RS.

## Case presentation

An 8-year-old right-handed girl with moderate intellectual disability presented with new-onset seizures triggered by micturition. She had spontaneous generalized tonic-clonic seizures of unclear aetiology at 5 months of age. Subsequently, she took anti-seizure medicines regularly, and had been seizure-free after drug discontinuation for 4 years. Her family history was unremarkable. She was delivered via caesarean section because of breech presentation, and her development was moderately delayed. She did not walk without assistance until the age of 3, and she started to use words at the age of 5. She had received speech therapy and physical therapy.

At the age of 7, she developed seizures that consistently occurred immediately on micturition, at a frequency of approximately seven or eight times per day. Each seizure was induced by micturition, regardless of whether she was in the seated or standing posture. Defecation did not trigger the episodes. When each episode started, she would initially stop talking, and this was followed by staring, leftward deviation of the head and eyes, axial and arm tonic posturing and extension of the upper extremities as if to hug someone, and brief rhythmic jerking of the upper body for a few seconds. The entire episode lasted for 10 to 15 s with impaired awareness, and she returned normal afterwards.

**Fig. 1:**
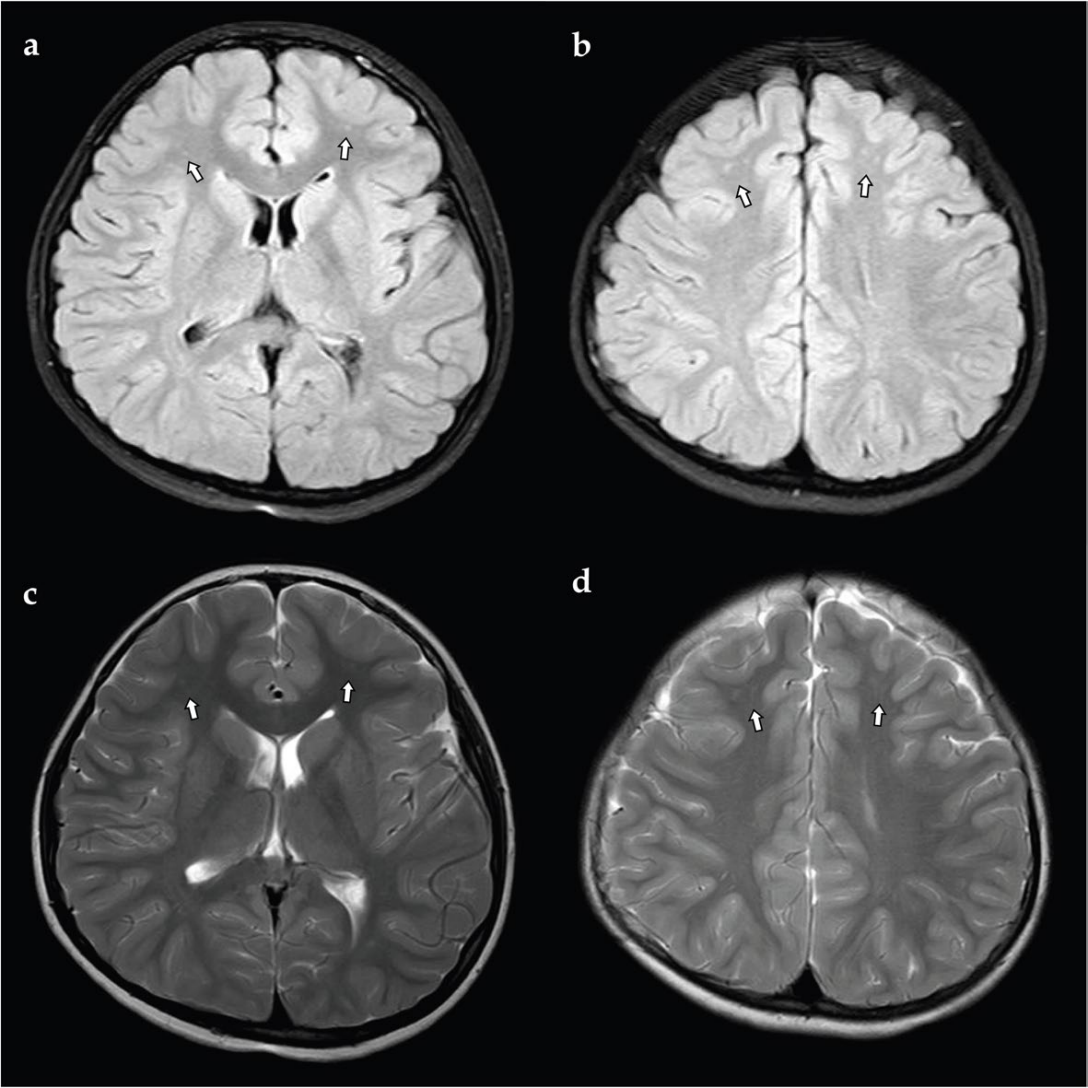
Cranial magnetic resonance imaging (MRI) showed scattered foci of long T1 signal in the bilateral inferior frontal cortex (arrows in **a**, **b**) and high signal on T2-weighted FLAIR (arrows in **c**, **d**)

**Fig. 2:**
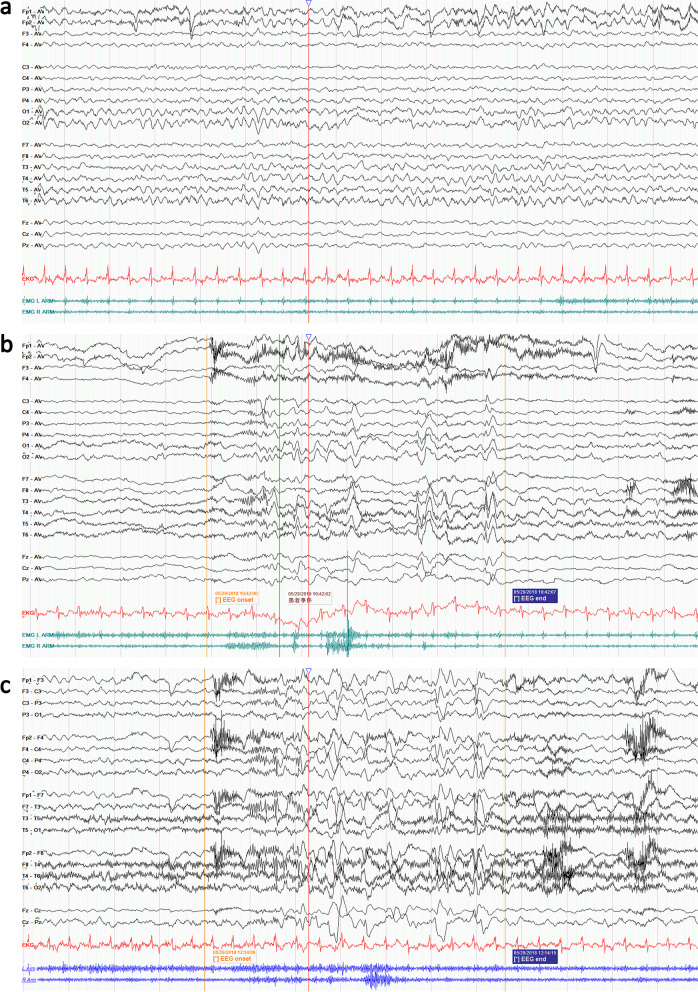
Scalp EEG recording of the present case. **a** Interictal EEG showed 5–6 Hz intermittent slow-wave background activity. **b** Ictal EEG showed low-voltage fast waves without clear focal features, followed by frontal dominant rhythmic multiple spikes in the average montage. **c** Ictal EEG showed diffuse low-amplitude fast waves in double banana montage. Red waves show the electrocardiogram signal, green waves and blue waves show the electromyography signal. The onset and end of the seizures are marked by yellow vertical lines. The red vertical lines represent the position corresponding to monitoring video playback and EEG. (Sensitivity at 10 µV/mm, LFF at 0.5 Hz, and HFF at 70 Hz)

Cranial magnetic resonance imaging showed hydrocephalus with several focal cerebral white matter lesions of unknown etiology (Fig. 1). The results of Holter monitoring and routine laboratory tests, such as complete blood cell count, glucose, electrolytes, liver function, urinalysis, and cerebrospinal fluid examination, were within the normal range. We recorded three typical seizures with video EEG monitoring, and all were induced by micturition. The seizures occurred a few seconds after micturition had begun, and the patient continued to urinate during the seizure. Scalp inter-ictal EEG showed a frontal dominant rhythm of 5–6 Hz (Fig. 2a). Ictal EEG showed low-voltage fast activity persisting 2 to 3 s without clear focal features, which then evolved into 3–4 Hz high-voltage irregular slow waves, and the post-ictal phase showed a normalized background (Fig. 2b and c). On the basis of clinical and EEG findings, the patient was diagnosed with RS induced by micturition. Valproic acid treatment was started, but the seizures were not controlled. After adding levetiracetam, the frequency of seizures was decreased to once to twice per month.

## Literature review

We searched the databases PubMed (Medline) for articles published up to January 2022 with search terms “micturition reflex seizure”, “reflex seizure induced by micturition”, and “micturition induced seizure”, and reviewed all the articles identified and their references. Articles written in English and Chinese were retrieved for further review and discussion; however, publications in other languages, including an article in Italian and another one in Japanese, were not included. We identified 13 previous cases combined with the present case to summarize the clinical and EEG characteristics of RS induced by micturition. The detailed clinical features are given in Table 1.

**Table 1:** Reflex seizures induced by micturition

Reference	Age at onset (years)/Gender	Trigger	Spontaneous seizure	Intellectual disability	Semiology features	Brain imaging	Inter-ictal EEG	Ictal EEG	Anti-seizure medicine	Outcome
Rathore [2]	3/M	Micturition	–	–	Forward flexion of the upper trunk and extension of the upper extremities	–	–	Beta burst at the Cz electrode	/	/
Viswanathan [3]	4/M	Micturition	+	+	Slow neck flexion, eye deviation to the right and posturing of the upper limbs	MRI: normal; Inter-ictal MEG: equivalent current dipoles clustered in the left midline posterior parietal region	Multiple foci	Fast activity in the midline centroparietal leads that culminated in the bilateral centroparietal region	Lamotrigine and valproate	Complete cessation
Zivin [4]	13/M	Micturition	–	–	Stiffening, lack of awareness and automatic activity	–	Right temporal foci	Slow waves and irregular dysrhythmic pattern in all the leads	Primidone	Remission
Bourgeois [5]	5/M	Micturition and immersion of feet in hot water	+	+	Sensation of pins and needles in the buttocks, staring, drooling, vocalization and fall	MRI: Chiari Type I malformation	Multiple foci	Onset in Cz	Carbamazepine and valproate	Partially controlled
Higuchi [6]	6/F	Micturition and defecation	+	+	Extension of the arms and rhythmic jerking in a conscious state	–	Spike-and-wave activity on central electrode recording	Rhythmic theta waves at the central electrodes	Clobazam and phenytoin	
Yang [7]	12/M	Micturition	–	+	Forward flexion of the upper trunk and eye deviation to the right	–	Bilateral multiple foci	Unclear activity	Oxcarbazepine	Remission
Okumura [8]	8/F	Micturition	+	_	Speech arrest, vocalization and extension of the upper extremities with preservation of consciousness	MRI: normal Subtraction ictal SPECT: mesial frontal	Normal	Fast waves without clear focal features followed by frontal dominant rhythmic multiple spikes	Phenytoin	Remission
Seth [9]	9/F	Micturition	–	–	Sense of fear, loss of awareness, tonic posturing and automatism	–	Possible left frontal slowing	Unclear activity	Lamotrigine	Remission
Whitney [10]	11/F	Micturition	+	+	Head and eye deviation to the right, flexed dystonic posturing of the limbs and eventual generalized tonic-clonic seizures	–	Multiple foci	Rhythmic theta activity in Cz followed by generalized activity	Lacosamide and clobazam	1.5 months without seizures
Glass [11]	10/F	Micturition and prayer	+	+	Staring, deviation of the eyes and head to the left and rhythmic clonic activity of both arms	MRI: normal Ictal SPECT: hyperperfusion in the anterior cingulate gyrus and anterolateral right frontal lobe	/	20–22 Hz rhythmic epileptiform activity at Cz, and spread to the bilateral frontal regions	Phenobarbital, valproic acid, clonazepam, topiramate, lamotrigine, clobazam, and ketogenic diet	Refractory
Cvetkovska [12]	9/M	Micturition and getting startled	+	Borderline	Tonic posturing, and occasional loss of body tone and fall	MRI: Focal cortical dysplasia in the right middle frontal gyrus	Focal rhythmic epileptiform discharges at F4/Fp2/Fz	Fast-spike discharges over the right lateral frontal region followed by generalized discharges	/	/
Rho YI [13]	7/M	Micturition	+	+	Hand automatism, secondarily generalized tonic posture and loss of consciousness	–	Generalized burst of spikes and slow complex waves, predominantly on both frontocentral areas	Onset from the left frontotemporal region and spread to the right frontotemporal region	Valproate sodium	Remission
Casciato [14]	18/M	Micturition	+	–	Genital pain followed by manipulation with preserved awareness; head deviation to the right and arm/truncal asymmetric tonic posturing, followed by bilateral tonic-clonic seizures	–	Spiking over the left fronto-temporal regions	Onset of rhythmical delta activity evolving into sharp waves over the midline involving the central-parietal and left fronto-temporal channels, with the recruiting rhythm evolving into a bilateral tonic-clonic seizure	Lacosamide	Seizure free for 3 months
Present case	8/F	Micturition	+	+	Speech arrest, deviation of the head and eyes to the left, tonic posturing, brief jerking and impaired awareness	MRI: Hydrocephalus	Generalized slowing background	Fast waves without clear focal features	Valproate sodium and levetiracetam	Partially controlled

Micturition-induced RS was most frequently recorded during the first decade of life (10 cases, 71.4%), with an age range of 3 to 18 years (mean age, 7.86 years). The 14 patients included 8 males (57.1%) and 6 females (42.9%). Micturition was the only trigger in 10 patients (71.4%), while a combination of triggers (apart from micturition) was reported in the remaining studies, for example, placing feet in hot water, defecation, praying, and getting startled. Ten patients (71.4%) experienced both provoked and spontaneous seizures. In addition, 10 of the 14 (71.4%) patients had normal structural neuroimaging results, and eight (57.1%) patients reported mental development delay. None reported a family history of seizures.

The seizure manifestations of the 14 patients were variable, but they all had seizures with focal onset. The seizures progressed to bilateral tonic-clonic seizures in two patients. With regard to the seizure semiology, posturing of the upper limbs (10 patients, 71.4%) was the most frequent presentation, followed by head/eye deviation (5 patients, 35.7%) and automatism (3 patients, 21.4%). In 8 patients (57.1%), awareness was preserved, whereas the others experienced a loss of consciousness. The inter-ictal EEG findings were unremarkable. In six reported cases (42.9%), ictal EEG indicated an epileptogenic focus in the midline region, while there were no clear focal features in five cases, including the case reported here.

Treatment with anti-seizure medication was documented in detail in 12 patients (85.7%): 6 with a monotherapy and the other 6 with a polytherapy. Valproate was used in five cases, lamotrigine in three, clobazam in three, and lacosamide in two. Six of the 14 cases (50%) became seizure-free, while one case was described as having refractory epilepsy.

## Discussion

In this article, we reported an 8-year-old pediatric patient with micturition-induced reflex epilepsy. So far, only a dozen cases of micturition-induced RS have been reported in literature. Based on a review of all the published cases [2–14], the main features of micturition-induced RS include: (1) a rather low prevalence, with an onset predominantly in childhood, but affecting both sexes equally; (2) common co-existence of developmental delay in cases; (3) concomitant occurrence of spontaneous seizures in nearly 70% of these patients, often preceding the micturition-induced RS; (4) the most common features of clinical semiology include, in a descending order of frequency, posturing of the upper limbs, deviation of the head and eyes, automatism, and a lack of awareness; (5) the true epileptic focus is not clearly identified, but the onset is likely to be from the midline region; and (6) prognosis generally satisfactory, as half of the cases achieved adequate seizure control with anti-seizure medication.

RS induced by micturition seems to have a young age of onset, except one case who showed onset age of 18 years. In contrast, RS associated with other triggers seems to typically have an onset in the second decade of life [15]. The majority of patients with RS induced by micturition present normal structural neuroimaging features, but a common documented history of developmental delay. This suggests that the micturition-induced seizures may be associated with brain immaturity as well as preexisting neurodevelopmental abnormalities.

In literature, nine cases were reported to have spontaneous seizures preceding the micturition-induced RS [3, 5, 6, 8, 10–14]. Previous studies showed that the majority of patients with RS also suffer from spontaneous seizures, and over 21% of the patients with idiopathic generalized epilepsy experience concurrent RS [16]. Some investigators proposed that RS and spontaneous seizures are two extremities of a conceptual continuum [17]. Changes in structural and functional brain networks occur after spontaneous seizures, resulting in hyperexcitability in certain brain areas, which would become more susceptible to epileptic discharges upon stimulation from a particular sensory, cognitive or motor stimulus [18]. In addition, there were three cases who experienced a seizure-free period from spontaneous seizures to RS, just like our case presented here [5, 11, 13]. This phenomenon has been rarely studied, and the underlying mechanism requires further investigation.

Glass et al. [11] performed ictal single-photon emission computed tomography (SPECT) within 5 s of seizure onset and revealed hyperperfusion in the anterior cingulate gyrus and the right anterolateral frontal lobe. Another ictal SPECT study revealed significantly increased perfusion in the mesial frontal regions [8]. These areas were close to or directly overlapping with the areas activated by urinary function [19]. Similarly, Cvetkovska and colleagues [12] reported focal cortical dysplasia in the right middle frontal gyrus, which overlapped with the brain regions activated by urinary function. Based on these findings, we speculate that the mechanism of micturition-induced RS may involve excessive excitation of regions that participate in micturition, as these regions would be more susceptible to epileptic discharges triggered by the physiological voiding process and, therefore, more prone to seizures during urination.

The exact epileptic focus of micturition-induced RS has not been clearly identified. Several reported cases assessed by ictal EEG showed a central epileptogenic focus [2, 3, 5, 6, 10, 11, 14], while others had no clear focal features. The semiology of micturition-induced RS is characterized by posturing of the upper limbs, deviation of the head and eyes, and automatism, which indicate the involvement of a common neural pathway. As a complex process, micturition is regulated by multiple levels of the central and peripheral nervous systems. The superomedial part of the frontal lobe and the anterior part of the cingulate gyrus control micturition [19]. The supplementary motor area (SMA) is activated during contraction of the pelvic floor muscles, which produces seizure activity, such as tonic posturing involving unilateral or bilateral extremities [20]. In the present study, although an unclear epileptic focus was identified by ictal EEG, we speculate that the SMA is involved in epileptic networks due to the shared clinical features of the reported cases.

## Conclusions

In conclusion, we report a case of RS-induced micturition with a focal initial EEG pattern. A review of all the reported cases so far indicate that the precise epileptic focus has not been clearly identified, but the onset is probably from the midline region. Future in-depth studies using advanced electrophysiology data acquisition and analysis techniques are needed to confirm these findings.

## Data Availability

The datasets used or analyzed in the present study are available from the corresponding author upon reasonable request.

## Abbreviations

EEG: Electroencephalogram
RS: Reflex seizures
SMA: Supplementary motor area
